# Development and contextual analysis of a person-centered professional practice model in a home care service in French-speaking Switzerland

**DOI:** 10.3389/frhs.2025.1566997

**Published:** 2025-06-17

**Authors:** Cedric Mabire, Sandra Panchaud, Jessica Wey, Justine Wicht

**Affiliations:** ^1^ Institute of Higher Education and Research in Healthcare-IUFRS, Lausanne University Hospital, University of Lausanne, Lausanne, Switzerland; ^2^ Pays d’Enhaut Health Network, Château d’Oex, Switzerland; ^3^ Sarine Health Network, Villars-sur-Glâne, Switzerland

**Keywords:** professional practice model, person-centered practice, home care services, implementation science, Switzerland

## Abstract

**Background:**

The Swiss healthcare system faces increasing challenges with an aging population and rising prevalence of chronic conditions, necessitating better-coordinated care delivery, particularly in home care settings.

**Objectives:**

This study aimed to develop (objective 1) and conduct a contextual analysis for implementation (objective 2) of a person-centered professional practice model for home care services in French-speaking Switzerland.

**Methods:**

A multi-method approach was used. For objective 1, concept mapping with 157 healthcare professionals (86% response rate) was conducted to develop the model. For objective 2, a contextual analysis was guided by the Intervention Mapping framework, involving focus groups with stakeholders (*n* = 14) and field validation with frontline staff (*n* = 6). Data analysis included both quantitative and qualitative methods.

**Results:**

The concept mapping process identified 13 core values rated on importance (scale 1–5), with health promotion scoring highest (4.4) and interprofessionalism lowest (3.7). Implementation analysis revealed key facilitators including leadership support (83% agreement) and barriers such as linguistic/cultural differences. Eight implementation strategies were identified and validated through a Delphi process, including continuous training (67% strong agreement) and safety culture promotion (83% strong agreement).

**Conclusions:**

The study demonstrates that developing and implementing a person-centered professional practice model is feasible in home care settings when supported by strong leadership commitment and structured implementation strategies. The model's alignment with the Person-centred Practice Framework of McCance and McCormack provides theoretical validation while offering practical guidance for implementation.

## Introduction

The Swiss healthcare system faces significant challenges due changing patterns of illness and functional limitations rather than demographic aging alone. While, by 2050, the number of people aged 60 and above is projected to double to 2.1 billion (1), research by Reinhardt (2) demonstrates that aging itself is not the primary driver of healthcare utilization. Instead, it is the increased prevalence of functional limitations and chronic conditions that directly drives home care demand (3). Switzerland mirrors this trend, experiencing a concurrent rise in non-communicable diseases (NCDs) and associated functional limitations. While these conditions are more prevalent in the growing older population segment (projected to reach 30% aged 65+ by 2050 (4), multimorbidity affects all age groups and is increasingly common, with estimates suggesting over 60% of those aged 65+ have multiple chronic conditions (5). This complex health profile, primarily driven by the burden of functional limitations and multimorbidity rather than age alone, underscores the limitations of a healthcare system historically geared towards acute care, leading to fragmentation and poor coordination (6). This fragmentation contributes to adverse health outcomes, increased healthcare expenditure, and a reduced quality of life, particularly for older adults with complex care needs (3).

In response, integrated care networks have been developing in Switzerland since the 1990s, evolving from primarily physician-centric models to encompass a broader spectrum of healthcare providers, including essential home care services (6). These networks strive to deliver comprehensive somatic and psychiatric care through multidisciplinary collaboration (7), addressing the intricate needs of individuals with multiple health conditions. However, the rapid expansion of these networks has often proceeded without clearly defined objectives and standardized implementation strategies, potentially creating a misalignment between system-level objectives and the professional values of healthcare practitioners (8). This ambiguity can foster confusion, resistance to change, and ultimately impede the successful implementation of integrated care initiatives (9). Professionals may perceive standardized protocols as a constraint on their professional autonomy and their capacity to deliver personalized care (10).

In Switzerland, home care services operate within a complex system of governance and financing (11). These services are regulated at the cantonal level (i.e., Swiss state or provincial), creating significant regional variations in organization and delivery. The canton plays a crucial role in this system by providing partial funding, establishing regulatory frameworks for service standards, approving organizational structures, conducting quality assessments, and bridging national policies with local implementation. Home care is primarily funded through a mixed system: mandatory health insurance covers nursing care prescribed by physicians, while the cantons, municipalities, and patients finance additional services themselves. The prescription process typically begins with a physician's order, followed by a needs assessment that determines the scope and intensity of services. The Sarine Health Network, where this study was conducted, operates within the canton of Fribourg and serves approximately twenty-eight municipalities with a population of 95,000 residents. Its distinctive feature is a decentralized organizational structure with six geographical branches plus a coordination center, all operating under a unified management but with significant operational autonomy. This district-level governance structure directly influences our operational capabilities, resource allocation, and strategic priorities, shaping which services we must provide and establishing quality standards we must meet. This structure, while allowing for local adaptation, has contributed to the fragmentation challenges that our research aims to address through a unified professional practice model.

Implementing integrated care within Switzerland's federalist structure necessitates a well-defined framework that promotes a shared vision and clear role distribution across the cantons. This is especially pertinent given the variations in resources and development across different cantons (12). Professional Practice Models (PPMs) have demonstrated their value in clarifying organizational values and missions, while simultaneously enhancing employee satisfaction and, importantly, improving patient outcomes (13). PPMs can bridge the gap between national-level strategies and local implementation, ensuring that the principles of integrated care are effectively translated into practice at the organizational level (14). Within this context, person-centered practice emerges as a critical approach to address these challenges, particularly within home care settings. Person-centered practice prioritizes individual needs, preferences, and goals, aligning with the core values of many healthcare professionals and cultivating a sense of partnership between patients and providers (15). Within home care, this approach is particularly valuable, as it enables care delivery within the patient's familiar environment, promoting autonomy and dignity (16). Furthermore, the integration of technology within home care, such as telehealth and remote monitoring, can further enhance Person-centered practice by facilitating continuous communication, support, and proactive care management (17).

Person-centered practice, as described by McCormack et al. (18), emphasizes treating individuals with dignity, compassion, and respect, while considering their personal experiences, values, and preferences in healthcare decision-making. This approach offers several benefits, including restoring meaning and coherence in the work environment (19), enhancing care consistency among health professionals, improving patient outcomes (20), bridging the gap between organizational goals and professional values, and adapting to the complex needs of home care patients (21, 22).

The integration of person-centered practice principles into a PPM for home care services offers a multifaceted solution to the challenges posed by Switzerland's evolving healthcare landscape (23). This approach has the potential to address several critical aspects simultaneously: it can enhance the quality of patient care, improve the work experience and job satisfaction of healthcare professionals, and clarify the values and mission of home care services within broader integrated care networks. By focusing on person-centered practice, this PPM can foster better coordination and continuity of care for patients with complex needs (24), while also strengthening the professional identity of home care staff (23). Moreover, it provides a framework for promoting consistency in care delivery across different providers, ultimately contributing to the broader implementation of person-centered practice principles throughout the Swiss healthcare system. This holistic approach not only aims to improve patient outcomes but also to restore meaning and coherence in the healthcare professionals' work environment, potentially leading to better staff retention and a more resilient healthcare workforce.

Within our specific home care network in Switzerland, these challenges manifest as fragmentation of care delivery across geographically dispersed teams. With multiple branches operating across different locations, inconsistent approaches to care have emerged, leading to discontinuities in service provision and potential variations in care quality. The absence of a unified professional practice framework has contributed to siloed operations, where teams develop branch-specific practices rather than implementing a cohesive, network-wide approach to person-centered practice. This geographical and operational dispersion presents unique challenges for maintaining consistent values and approaches across all network components.

This study objectives are (1) to identify and structure the core professional values perceived by healthcare staff as fundamental to guiding the development of a person-centered PPM for home care services in French-speaking Switzerland, and (2) to understand and analyze the perceived determinants (barriers and facilitators) influencing the potential implementation of a person-centered practice model within home care services in French-speaking Switzerland. Specifically, our research targeted the fragmentation of care delivery across geographically dispersed teams in our home care network, by creating a unified professional framework that would establish consistent person-centered values and approaches across all network branches, providing a cohesive foundation for care delivery despite geographical dispersion.

## Methods

### Setting

The Sarine Health Network (RSS) was established on January 1st, 2016, in the Sarine district of Fribourg canton, Switzerland. Its primary mission is to facilitate care for vulnerable individuals while improving healthcare efficiency, cohesion, and cost-effectiveness. The RSS integrates seven distinct services: the Coordination Center, the nursing home commission, the commission for flat-rate allowances, the ambulance service, the Sarine Nursing Home, the Sarine Home Care Service (SASDS), and, since January 2023, the Sarine fire service. The organization's activities are guided by three core values: responsibility, professionalism, and respect.

This study specifically focused on two components of the RSS: the SASDS and the Coordination Center, both operating under a unified Nursing Care Management. These two services play a crucial role in coordinating and delivering home care within the district.

The Coordination Center, composed of a manager and nurses, is responsible for processing new healthcare requests from the Sarine district population, managing family caregiver allowances, and overseeing administrative staff who maintain direct patient contact.

The SASDS provides home care services through seven geographical branches, each led by a manager who oversees an interprofessional team of nurses, healthcare assistants, and nursing aids. The service is further supported by an occupational therapist, dieticians, and a specialized wound care nurse. Together, these two services employ 183 staff members, providing care to approximately 2,300 clients annually.

#### Objective 1: development of the professional practice model

**Design:** For the development phase, the study employed a cross-sectional multi-method design following the concept mapping process described by Kane and Trochim (25).

**Sample:** The target population consisted of all employees from both the SASDS and the Coordination Center (*N* = 183) who met the inclusion criteria of current employment and French language proficiency. Following Kane and Trochim's concept mapping methodology, we selected a purposive subsample of 17 professionals from both the SASDS and the Coordination Center to participate in the detailed mapping activities. The selection ensured representation across all service roles, including clinical staff (seven nurses with one mental health specialist, four healthcare assistants, and two nursing aids), support services (one administrative staff member), specialized professionals (one occupational therapist and one dietician), and management (one branch manager). We determined this sample size based on previous concept mapping studies suggesting that 10–20 participants provide sufficient variety in perspectives while maintaining feasible group dynamics.

**Data collection**: The concept mapping process unfolded through six sequential steps (25). The preparation steps involved clarifying project objectives, defining the sample, and establishing a detailed schedule through a Gantt chart. Eight information meetings were conducted with stakeholders to ensure comprehensive understanding and engagement. The Concept Systems Incorporated software was utilized to develop demographic questions and the primary focus question (26).

During the generation steps (May 8–June 2, 2023), participants responded anonymously to the focus question: “Based on your role within SASDS, could you define what is important to you in providing services that meet patient/beneficiary needs?” Responses were collected through The Concept Systems Incorporated software, with participants having access to view previously recorded responses to avoid duplication. The project team subsequently refined the response set by removing duplicate and unclear statements.

The structuring steps involved the 17-member subsample participating in two-hour sessions in July 2023. During these sessions, participants individually sorted and categorized statements based on perceived similarities and rated each statement's importance on a 5-point Likert scale (1 = not important, 5 = extremely important). The representation stage utilized the Concept Systems Incorporated software to perform multidimensional scaling and hierarchical cluster analysis, generating concept maps that visualized the relationships between statements.

In the interpretation steps, the project team examined the concept maps and identified 15 core values. These findings were presented to SASDS management and branch managers for validation and refinement. An in-person focus group with 8 participants (managers) was conducted to validate the visual design, ensuring data triangulation through diverse stakeholder feedback. The utilization steps involved applying these results to guide subsequent implementation phases.

**Data analysis:** Data analysis was conducted using Concept Systems software (26), which is specifically designed for concept mapping methodology. The analysis process followed the standard steps of this approach. After data collection, the software was used to perform multidimensional scaling of the sorts completed by participants, generating a similarity matrix (27). This matrix was then subjected to hierarchical cluster analysis to group statements into broader concepts (25).

The software generated visual representations of the relationships between statements in the form of concept maps. These maps were collaboratively interpreted with participants to name clusters and identify structuring axes, ensuring a collective understanding of the studied issue (27). The software facilitated the creation of interactive concept maps and pattern matches.

Participants' ratings on predefined criteria were analyzed to provide complementary information on the relative importance of different identified concepts. The Concept Systems software allowed for the input of card sort piles and ratings from participants, which were then analyzed to produce visual representations of the data (27).

This analysis approach enabled the organization of disparate ideas, linking of similar thoughts, and equal consideration of contributions from numerous participants. The resulting concept maps illustrated group ideas and concerns, how ideas were related to one another in a multidimensional concept space, how ideas were organized into general concepts, and how concepts were rated in terms of criteria relevant to stakeholders (27).

#### Connection between concept mapping and intervention mapping

The two methodological approaches were directly connected, with concept mapping providing the foundation for intervention mapping. The concept mapping process from Objective 1 produced the core values and structural framework of our Professional Practice Model. These values then directly informed the intervention mapping in several ways: they provided the foundation for the needs assessment, helped define implementation objectives, guided the selection of theory-based methods, and served as evaluation criteria for implementation strategies. The 15 core values identified became the central content that needed to be implemented, with particular attention to the highest-rated values (health promotion and patient-centered approach). The visualization created during concept mapping was used as a communication tool during intervention mapping focus groups to maintain conceptual consistency. Additionally, areas that received lower ratings in concept mapping (particularly interprofessionalism) were prioritized for targeted implementation strategies during intervention mapping.

#### Objective 2: contextual analysis for the implementation of person-centered PPM

**Design:** The contextual analysis for the implementation phase employed the Intervention Mapping (IM) framework (28). While this framework was originally developed for health promotion programs, we adapted it for our professional practice model development because of its structured, stepwise approach that emphasizes stakeholder involvement and theoretical grounding. We found its systematic approach to development, implementation planning, and evaluation particularly valuable for structuring our work in the complex home care environment, despite this representing an adaptation from its original purpose. The framework was utilized with a participatory research approach to ensure alignment with population needs and contextual factors (29).

**Sample:** The contextual analysis process involved two groups strategically formed to ensure continuity from the development phase. The resource group (*n* = 3) consisted of the Nursing Care Director, the same Clinical Nurse Specialist (CNS) who had led the concept mapping phase, and a newly appointed SASDS head nurse. The stakeholder group (*n* = 6) was expanded from the initial concept mapping participants to include the Coordination Center manager, all six branch managers (including the branch manager who participated in the concept mapping), and the same occupational therapist (MScHS) from the development phase. This composition maintained key participants from the first phase while broadening representation across the organization's leadership structure to facilitate implementation.

**Data collection:** Implementation followed four key stages of the IM framework (28). The needs assessment stage consisted of three two-hour focus groups with the stakeholder group over a two-month period. During these sessions, semi-structured focus groups based on the Consolidated Framework for Implementation Research (CFIR) identified implementation barriers and facilitators (30). Focus group discussions were guided by a pre-defined interview guide developed from the CFIR domains.

The objectives definition stage involved two additional focus groups where executive nurses shared their understanding and vision of person-centered practice, and stakeholders identified existing facilitators that could support the implementation of the professional practice model developed in phase 1. The theory-based methods selection stage examined current care delivery practices and implementation strategies already established within the organization that aligned with person-centered practice principles.

The intervention design stage utilized a modified Delphi method to achieve consensus on implementation strategies. This process involved two rounds: first, a focus group to discuss and refine the strategies, followed by stakeholders rating identified implementation strategies using a 5-point Likert scale questionnaire. For validation purposes, we conducted one additional focus groups with 6 field staff members (nurses, healthcare assistants, and nursing aids) who had not participated in previous stages. These sessions included both structured discussions and individual questionnaires with Likert scale ratings (0–5) and open-ended comments sections. The sequential nature of data collection allowed for continuous refinement of implementation strategies based on stakeholder feedback and field validation.

**Data analysis**: The qualitative analysis employed a rapid analysis approach based on Nevedal's method, incorporating both deductive coding using the CFIR Codebook and inductive coding for emerging themes (31). Codes were weighted on a scale from −2 to +2, and data triangulation was performed with the CNS and research team. Quantitative analysis of questionnaire responses utilized Excel-based analysis, including response distribution and percentage calculations.

### Ethical considerations

The requirement for ethical approval for the studies involving humans was waived by the Reseau Sante Sarine (RSS) Board Direction. This decision was made in accordance with the RSS's specific local institutional guidelines. These internal guidelines stipulate that service development projects and professional practice evaluations—which primarily involve an institution's own staff perspectives and do not directly impinge on patient interventions, nor make use of sensitive, identifiable patient health data requiring review by an external cantonal committee under Swiss human research legislation—fall under the ethical oversight of the RSS Board of Directors. This study, aimed at developing a professional practice model through the input of healthcare professionals regarding their work and service organisation, was classified as such a project.

Despite this specific authorisation pathway, the study was conducted in strict adherence to all applicable organisational ethical guidelines and fundamental international ethical principles, including those outlined in the Declaration of Helsinki. Voluntary participation and the right to withdraw from the study at any time were ensured for all participants. Information meetings were conducted to ensure that all potential participants clearly understood the study objectives, procedures, and their rights before written informed consent was obtained for all data collection activities. Anonymity was maintained during the concept mapping phase. Focus group recordings were used solely for the purpose of analysis, with explicit consent obtained from participants for their use; confidentiality was prioritised throughout all stages of data collection and analysis. To minimize potential fatigue and ensure minimal interference with professional responsibilities, sessions involving participants, such as focus groups and structuring activities, were limited to a maximum of two hours, with breaks provided as needed during longer activities.

## Results

### Objective 1: development of the professional practice model

The concept mapping process achieved a high participation rate of 86%. During the idea generation phase, participants responded to the focal question about defining important elements for meeting patient needs in their service delivery. This process yielded 325 initial statements, which the research team systematically refined through consensus. After eliminating duplicates, synonyms, and unclear statements, 111 unique statements remained for further analysis. These statements underwent sorting and rating during structured sessions.

**Figure 1:**
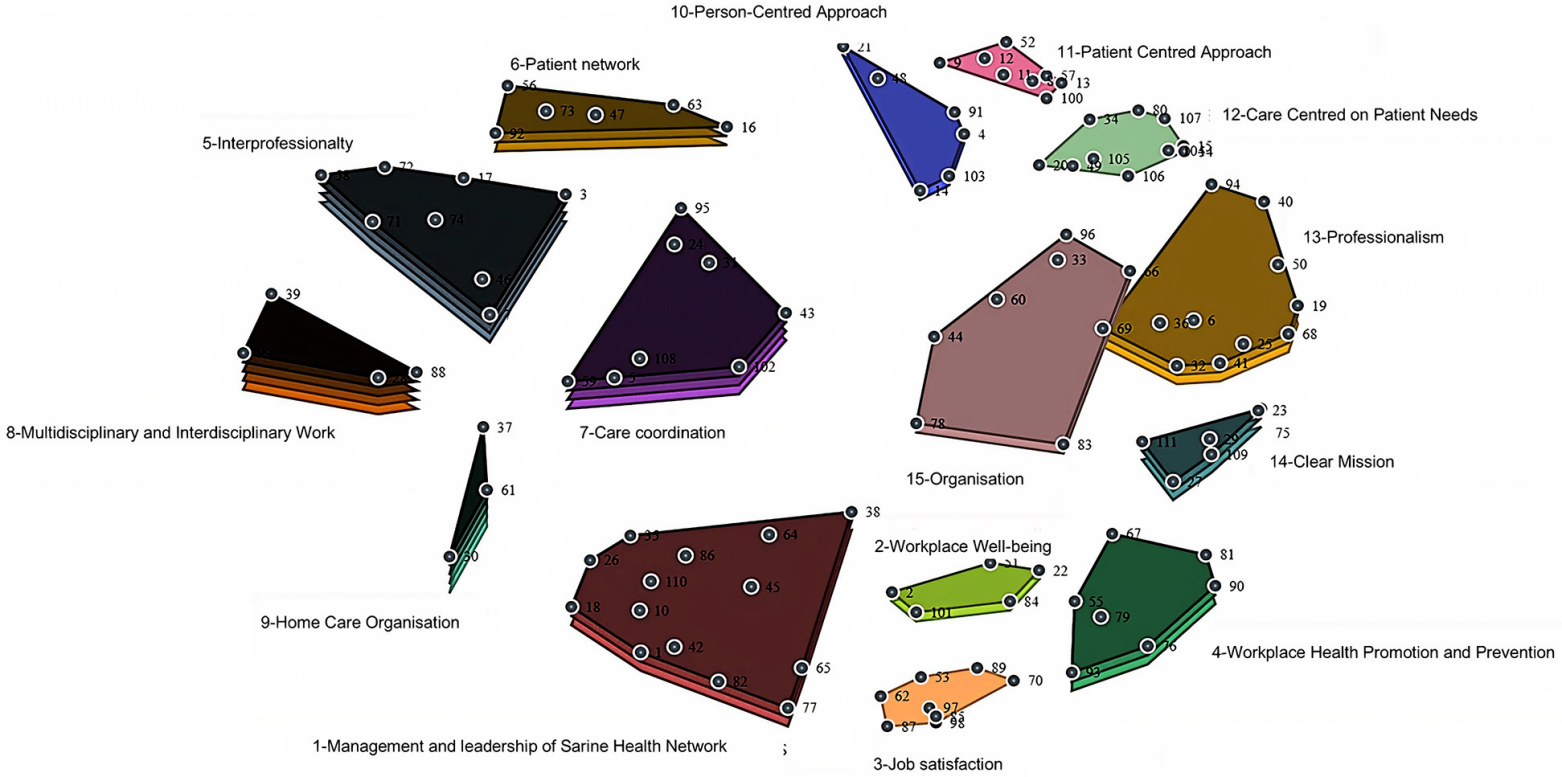
Flow chart.

**Figure 2:**
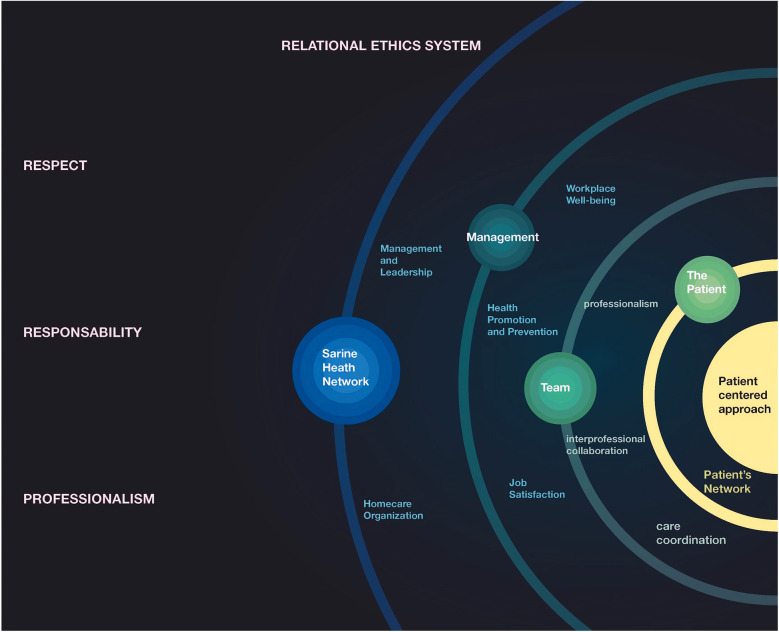
Cluster concept Map generated from participant data analysis, showing relationships between potential components of a professional practice model at SASDS.

The subsequent analysis initially identified 15 distinct values, which were later refined to 14 core values through an iterative stakeholder validation process. This refinement process revealed ten key attributes that crossed multiple values: training, staff autonomy, patient autonomy, professional positioning, equity, quality of care, empathy, holistic approach, communication, and collaboration. These attributes served to clarify and enhance the definition of the core values of the professional practice model (Figure 1). Figure 2 presents the cluster concept map generated directly from the multidimensional scaling and hierarchical cluster analysis of participant sorting data. It visually represents the statistical proximity and relationships between the identified value clusters as perceived by the study participants, forming the empirical basis for the final model.

The concept mapping process identified 14 core values that form the foundation of our Professional Practice Model. Below, we present these values in descending order based on their importance ratings (1–5 scale), from highest to lowest rated.

Health promotion and prevention emerged as the highest-rated value at 4.4, emphasizing the importance of maintaining a healthy work environment while supporting staff autonomy in healthcare delivery. Participants described this value as encompassing approaches to both staff and patient well-being. Importantly, participants viewed health promotion as foundational to effective care coordination across our geographically dispersed network. When different branches consistently prioritize health promotion, they create a shared starting point for care planning that helps overcome fragmentation. As one participant noted, ‘A unified approach to supporting patient self-care creates natural coordination points between different providers and services.

The patient-centered approach emerged as the second highest-rated value (4.3), incorporating holistic care, empathy, and patient autonomy as key attributes. This value emphasized the active consideration of patient needs and preferences in care planning and delivery, positioning patients as central decision-makers in their care journey.

Job satisfaction scored 4.2, demonstrating its significance in the organizational context. This value encompassed various elements contributing to employee contentment, including occupational health and safety measures, recognition of staff contributions, and active listening from management. A particular emphasis was placed on aligning institutional objectives with staff aspirations to create a harmonious work environment.

Professionalism (rated 4.1) integrated several crucial elements, including care quality, equity, and professional positioning. This value emphasized the importance of maintaining high standards while ensuring equitable care delivery across all patient populations.

Workplace well-being received a rating of 4.1, reflecting its critical role in maintaining organizational health. This value incorporated multiple dimensions, including service flexibility to adapt to changing needs, clear job descriptions to establish role boundaries, and adequate time allocation for patient care. The emphasis on professional qualifications within this value highlighted the organization's commitment to maintaining high standards of care delivery.

The SASDS organization value (rated 4.0) encompassed the structural and operational aspects necessary for effective home care delivery. This value focused on ensuring that organizational mechanisms and practices consistently support high-quality healthcare service provision.

The patient network value (rated 3.9) focused on developing and maintaining strong partnerships with patients, their families, and caregivers. This value recognized that effective care extends beyond direct patient interaction to include the broader support network essential for optimal health outcomes.

The management and leadership value, rated 3.8 out of 5 on the importance scale, emerged as a fundamental component focused on coordinating human and material resources. This value particularly emphasized the importance of training and development, highlighting the organization's commitment to continuous professional growth. It encompassed leadership abilities essential for motivating and guiding team members effectively.

Care coordination received a rating of 3.7, emphasizing the importance of integrated care management. This value particularly highlighted communication and collaboration as essential attributes for ensuring seamless care delivery across different providers and settings.

Although receiving the lowest rating (3.7), interprofessionalism was recognized as crucial for comprehensive care delivery. This value emphasized the importance of collaborative approaches across different health professionals and services, acknowledging that effective patient care requires integrated expertise from multiple disciplines.

Four additional values were incorporated to align with broader organizational principles. Respect was added as a transversal value at management's request, emphasizing fundamental human dignity and acceptance of others' rights and opinions. Similarly, responsibility was included to reflect professional commitment and reliability. Relational ethics was incorporated as a transversal value emphasizing the importance of interpersonal relationships, compassion, trust, and effective communication in healthcare delivery.

The Professional Practice Model was constructed by organizing the 14 identified values into a coherent framework that reflects their interrelationships and relative importance. As shown in Figure 2, health promotion and patient-centered approach form the central core of the model as the highest-rated values (4.4 and 4.3 respectively). These are surrounded by supporting values arranged according to their importance ratings. The three transversal values (respect, responsibility, and relational ethics) permeate all levels of the model.

**Figure 3:**
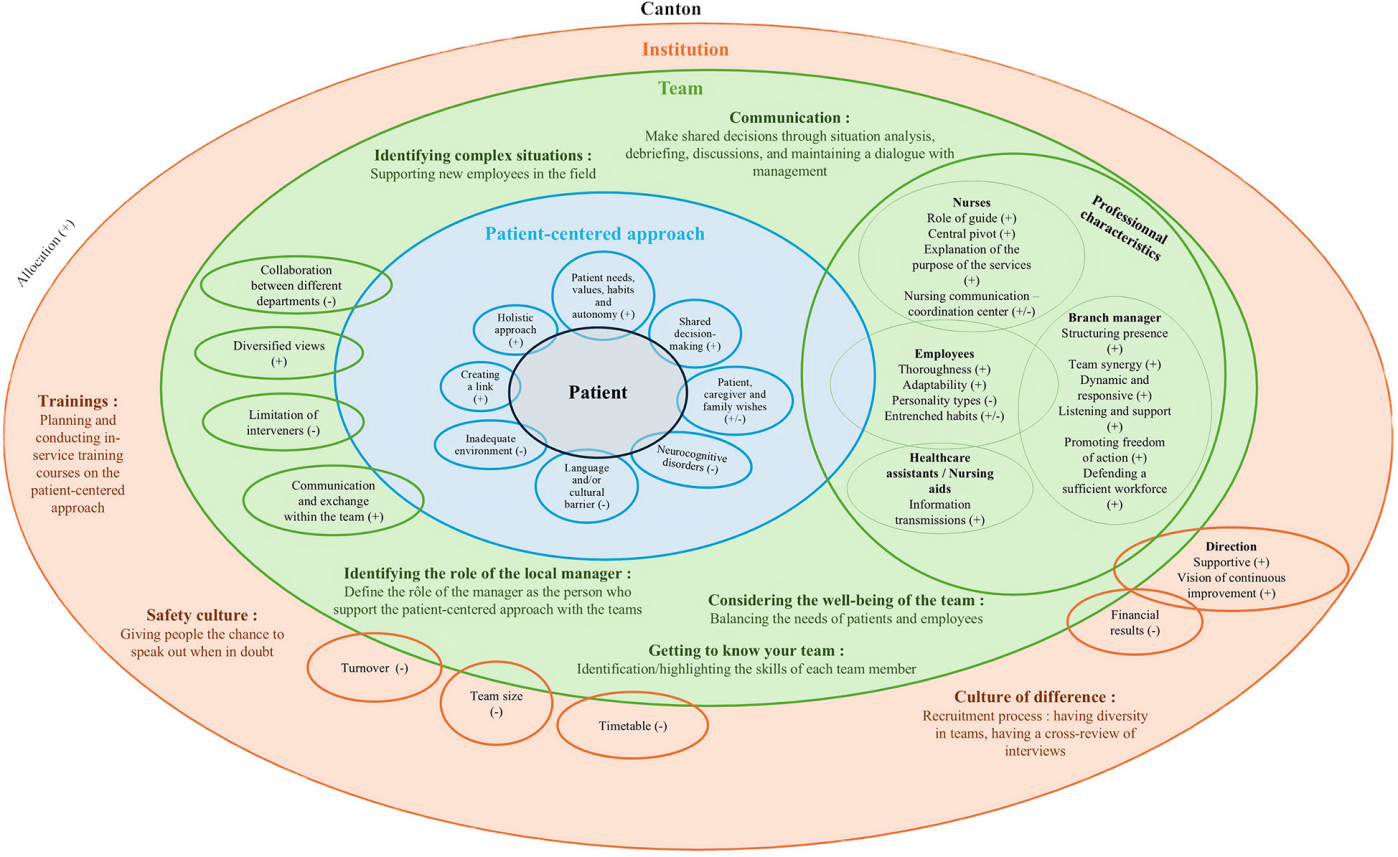
Synthesized visual representation of the SASDS professional practice model, showing the hierarchical organization of core values (based on importance ratings) and their interrelationships.

Based on the conceptual relationships identified in Figure 2 and incorporating the importance ratings assigned by participants, the final Professional Practice Model (PPM) was constructed and synthesized into a visual framework presented in Figure 3. Through focus group consultation, stakeholders selected the solar system design shown in Figure 3 as it effectively conveys the hierarchical organization and interconnected nature of the 14 core values within the final PPM framework. The design illustrates how these values interact and support each other in practice, creating a cohesive approach that can be implemented across all network branches.

The validation process not only confirmed the relevance and comprehensiveness of the identified values but also ensured their alignment with organizational objectives and RSS core values. Notably, the resulting model showed significant alignment with established theoretical frameworks, particularly the Person-centred Practice Framework of McCance and McCormack, providing additional validation of its theoretical underpinning (18).

### Objective 2: contextual analysis for the implementation of person-centered professional practice model

Analysis of the focus groups revealed several key determinants influencing the implementation process. The successful implementation of person-centered practice fundamentally depended on individualizing care approaches and engaging in active listening. A branch manager articulated this essential approach: “We identify their habits regarding care…there's also the notion of including the patient in their project. We identify objectives, a care plan. We accompany them in their goal, whether it's recovering autonomy, maintaining it, or promoting whatever is needed.” This perspective resonated consistently across both management and front-line staff perspectives.

Beyond individualized care, a holistic approach proved fundamental to implementation success, with branch managers particularly emphasizing the importance of considering social, familial, psychological, and economic dimensions in care delivery. However, the implementation process faced significant challenges, particularly regarding linguistic and cultural differences. While healthcare providers consistently strived to meet patient expectations, resource limitations often necessitated extended discussions to properly align values and expectations.

The implementation of person-centered practice proved particularly challenging for patients with cognitive impairments. Care teams frequently encountered complex situations where patients' expressed wishes conflicted with safety requirements. Both management and front-line staff consistently emphasized the critical nature of building therapeutic relationships, observing that trust development varied significantly among patients, with some forming connections quickly while others required extended time and regular visits to establish meaningful relationships.

Within this implementation context, patient support networks emerged as both facilitators and potential barriers to success. Family involvement proved especially crucial in decision-making processes, particularly for patients with cognitive impairments. One participant eloquently described this dynamic: “The idea is to have a therapeutic relationship with both the patient and their entire environment, and to co-create this relationship with all stakeholders—home care, physician, really all participants. We talk a lot about partnership…It's a co-construction of all objectives and interventions we're going to do together.”

The success of implementation relied heavily on team dynamics and professional relationships. Branch managers consistently emphasized the importance of thorough preparation for daily rounds, acknowledging the unique challenges each day presented. This perspective was captured by one manager who reflected: “We're in a constantly evolving world, we must always adapt for many things. It's also an adaptation that we must make, and all collaborators must constantly adapt, and I think it's important to keep this in mind. But everything revolves around the patient, keeping them at the center of our concerns.”

Within the team structure, reference nurses emerged as critical facilitators of implementation. These professionals served as essential information hubs, developing intervention plans aligned with patient wishes while coordinating information flow between healthcare assistants and nursing aids. Their effective communication with the coordination center proved particularly crucial in preventing unnecessary hospitalizations.

The role of leadership proved fundamental to implementation success, with branch managers positioning themselves as essential supporters of the person-centered approach. They provided structural presence and maintained team cohesion throughout the implementation process. The importance of responsive leadership was emphasized by one manager who noted: “It's important for me to hear team feedback. What do you need to have a patient-centered approach? What are your needs? Express them, and then we'll look at what we can implement.”

The broader organizational structure significantly influenced implementation in multiple ways. While management demonstrated strong support for continuous improvement, the implementation process revealed tensions between meeting patient needs and maintaining financial sustainability, particularly regarding billable hours. This dynamic was captured by one focus group participant who observed: “We have a management team right now that is supportive and encouraging,” while acknowledging the practical challenges of balancing care quality with operational constraints.

Throughout the implementation process, several key strategies emerged, already embedded within the organization's practices. The foundation of these strategies rested heavily on continuous professional development, which proved essential in strengthening multiple aspects of person-centered practice implementation. Training activities focused on enhancing understanding of patient needs, facilitating shared decision-making, and promoting holistic care approaches. Participants described the organization's approach to professional development, with one staff member noting, “Things are being done and developed, we provide training. Training is offered. Management is open to training.” This commitment was further validated through quantitative data, with 67% of stakeholders strongly agreeing they received adequate training, while the remaining 33% somewhat agreed.

**Figure 4:**
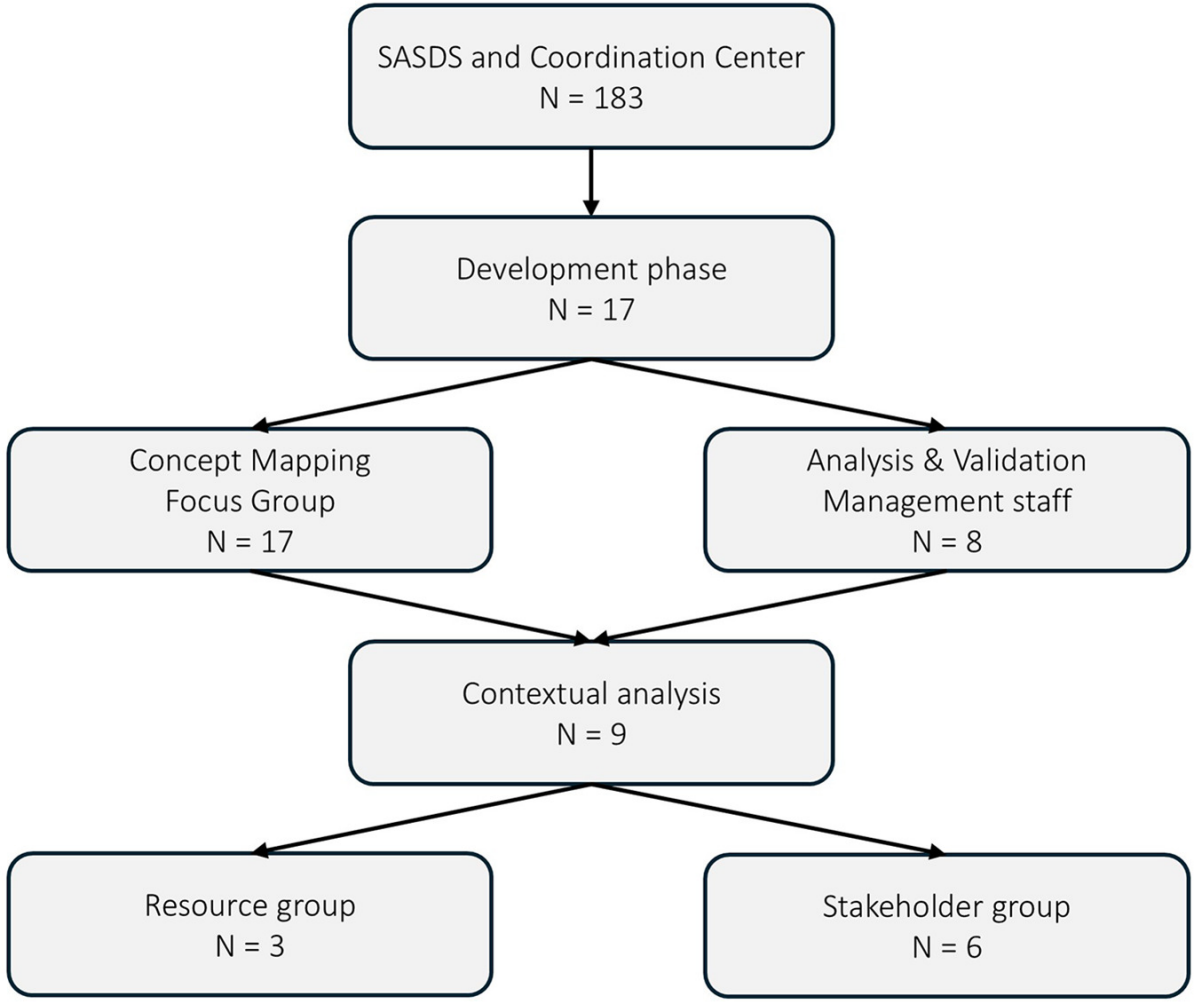
Determinants and implementation strategies of the SASDS professional practice model.

Building on this foundation of professional development, the organization implemented a strategic approach to recruitment that emphasized diversity in professional backgrounds and expertise (Figure 4). This approach represented a significant shift in hiring practices, as explained by one manager: “The recruitment approach is completely different now. Management's message is to hire based on the specific skills needed in our branch…When we post a position, we ask for experience because that's what we'll be missing. For healthcare assistants, we might request specific training…For nurses, we might look for cardiovascular expertise or other specialties we need.” This recruitment approach aimed to create teams with diverse professional skills.

The organization further supported implementation through robust shared decision-making processes. Communication strategies centered on systematic situation analysis, regular debriefing sessions, and continuous dialogue with management. These processes were designed to be inclusive and collaborative, as one participant described: “Situations are presented and everyone brings up the problems they encounter, everyone has a voice. Then there are exchanges about the situation. It can be brainstorming, the care plan evolves. Reference nurses take action or not.” However, this approach faced practical challenges, particularly regarding time constraints. Front-line staff expressed concerns about the limited time available for in-depth analysis, with one participant noting: “Time is limited, there's a lot at the start and then only 20–30 min remain for situation analysis, and that's short. And I feel that's what we need.”

To maximize the effectiveness of their diverse workforce, the organization developed a comprehensive team competency mapping approach. This mapping approach connected available skills with specific patient needs. The approach not only enhanced patient care delivery but also supported professional development and improved care coordination across teams.

Branch managers were involved in the implementation process as facilitators of the person-centered approach. While this strategy received mixed responses in the Delphi validation, quantitative data showed strong support from front-line staff, with 83% strongly agreeing that branch managers supported the person-centered approach, and 67% strongly agreeing about overall management support. However, some participants noted gaps between expectations and practical implementation realities.

The organization developed specific approaches for managing complex cases and supporting new staff members, recognizing the challenges these situations presented. A manager detailed this approach: “There's identification of situations requiring supervision regarding patient knowledge from referents…these patients are systematically paired multiple times until the person feels comfortable. It's not just once…We also make staff vigilant, so they don't find themselves failing. There's preventive work around this.” This strategy received strong validation, with 83% of stakeholders strongly agreeing that adequate support was provided for challenging cases.

Recognizing the interconnected nature of staff and patient well-being, the organization placed significant emphasis on maintaining a balance between these two aspects. This approach was reflected in one participant's observation: “We spend as much time and energy on work centered on staff as on patients…We're more focused on team well-being than patient well-being. While it should primarily be the reverse. Although one doesn't go without the other.”

The organization's commitment to safety and open communication was evident in its promotion of a culture where staff felt comfortable expressing doubts or concerns. A manager described this approach: “We asked if they thought they could tell us when they're not sure about what they're doing…They see there's always a positive reception from us…There's always a yes, I'll stop what I'm doing, and we'll discuss. And having this benevolence in the team means that nobody ever disturbs the other and we always manage to build together.” This emphasis on psychological safety received strong validation, with 83% of stakeholders strongly agreeing they could freely express concerns to both colleagues and managers.

Despite these comprehensive strategies, implementation faced several persistent challenges. The size of teams significantly impacted communication effectiveness, with larger teams experiencing more difficulties in information exchange. Staff turnover posed ongoing challenges to maintaining stable patient-provider relationships, while visit scheduling constraints and financial pressures created continuous tensions between meeting patient preferences and maintaining organizational efficiency.

Interprofessional collaboration emerged as a particularly significant challenge. As one participant observed: “The barriers include the network which can be extremely important with different services. Collaboration is sometimes difficult between services. Everyone works a little bit in their domain. There is little communication, I would say a lack of partnership between services.”

The implementation process benefited significantly from a favorable political context, including recent increases in staffing allocations. This external support was highlighted by a participant who noted: “Home care has been enjoying an absolutely incredible cantonal political context for 3–4 years. There has been an increase in allocated positions.” This positive political environment strengthened the organization's capacity to implement and sustain person-centered practice practices effectively.

## Discussion

This study of a home care network has yielded significant insights into both the development and context analysis for a Professional Practice Model (PPM). Through engagement with stakeholders across multiple care branches, our findings illuminate important considerations when developing a theoretical framework for potential application in home care settings.

When interpreting our results in the context of existing literature, in the decentralized context of home healthcare, where teams often operate in geographical and professional isolation, stakeholders identified that a unified Professional Practice Model could potentially serve as stabilizing force (7). Our contextual analysis suggests that such a model, if successfully implemented, might help bridge diverse practices across the network's branches. This potential unifying function would be particularly valuable in the home care context, where physical separation of teams can lead to divergent practices and approaches.

The model's potential for fostering cohesive care practices aligns with Slatyer et al.'s (32) findings in hospital settings, while suggesting its possible application to enhance integration within home care networks. Supporting this extension, Imhof et al.'s (33) research on Advanced Practice Nurses (APNs) in home care demonstrates how structured professional frameworks can enhance quality of life and health outcomes for community-dwelling older adults. While our study identified distinctive challenges within the home care environment, it also revealed opportunities for improving care coordination and practice standardization through the model's implementation.

The involvement of all stakeholders in the model's development, from frontline staff to management, proved crucial in establishing its legitimacy and applicability. The high response rate (86%) in the concept mapping phase suggests the strong engagement of staff across all levels, indicating a collective recognition of the need for a unifying framework. This broad participation helped ensure that the resulting model reflected the real-world experiences and needs of those delivering care, rather than merely representing a top-down theoretical construct. Including intended end-users in guideline development is a moral imperative and critical for addressing the right issues (34). Furthermore, as emphasized by Wiig et al. (35), involving diverse stakeholders, including patients, healthcare professionals, and managers, is crucial for creating resilient healthcare systems, which aligns with our observation of broad participation across all levels.

Critical analysis of our results reveals that a particularly noteworthy finding was the striking alignment between our developed PPM and the Person-centred Practice Framework of McCance and McCormack (18). This congruence emerged organically through the development process, rather than being deliberately engineered, lending additional credibility to both our model and the Person-centred Practice Framework of McCance and McCormack. The natural alignment suggests that the fundamental principles of person-centered practice resonate deeply with home care practitioners, regardless of their role or level within the organization.

This alignment manifested across multiple dimensions. The values identified through our concept mapping process, particularly those emphasizing patient autonomy, holistic care approaches, and professional competence, mirror the core components of McCance and McCormack's model (18). As Kitson et al. (36) highlight, these elements are crucial in creating a truly person-centered practice environment. Participants noted this connection in relation to the home care context and the direct nature of care delivery.

The congruence between our PPM and McCance and McCormack's model provided more than theoretical validation; it offered a robust framework for practical application across the network. By establishing a shared language and common vision for clinical outcomes that transcended individual branches, the model facilitated more coherent care delivery in our decentralized setting.

The hierarchical structure of our PPM, with clearly defined core values arranged by importance, provides a clear blueprint for implementing person-centered practice and directly addresses our primary aim of reducing care fragmentation across geographically dispersed teams by establishing value priorities while showing their interconnections. By providing a unified framework of professional values, the model creates a common language and shared priorities that transcend branch-specific practices. When all branches align their operations around the same core values—particularly the highest-rated health promotion and patient-centered approach—they naturally develop more consistent care approaches. This consistency helps bridge the geographical and operational gaps that previously led to fragmented care. The visual representation as a cohesive system further reinforces the interconnected nature of these values, encouraging practitioners to view their work as part of an integrated whole rather than isolated branch-specific activities. The four transversal values that permeate all levels ensure ethical and professional continuity across the entire network. This structured approach to professional practice provides the foundation for standardized yet flexible care delivery that maintains consistency while accommodating local context—essential for reducing fragmentation in a decentralized home care system. It is crucial to emphasize that this PPM is intended as a high-level conceptual framework designed to guide strategic direction, decision-making, and practice development by establishing shared values and priorities. It is not an operational blueprint dictating specific day-to-day procedures or protocols, which would need to be developed subsequently in alignment with this guiding framework.

Despite this alignment, our model does present distinct characteristics when compared to McCormack's model. Our approach contextualizes person-centered practice principles specifically within a decentralized home care network structure, addressing organizational challenges unique to this setting. The concept mapping methodology revealed specific value prioritization patterns in our context, particularly the relatively lower rating of interprofessionalism despite its recognized importance. Additionally, our framework integrates organizational values (responsibility, professionalism, respect) with person-centered principles, reflecting the specific cultural context of our Swiss home care network. These differences represent contextual adaptations to our specific operational environment rather than fundamental conceptual departures from McCance and McCormack's comprehensive model.

The contextual analysis phase of our study revealed further significant insights, particularly in how emerging strategies naturally aligned with McCance and McCormack's key dimensions of “The practice environment” and “Prerequisites”. This natural alignment between theoretical constructs and practical application strategies suggests that McCance and McCormack's model provides a particularly suitable framework for home care settings.

In examining the practice environment, our findings revealed that strategies of open communication and collaborative decision-making aligned closely with McCance and McCormack's framework of shared power and effective staff relationships. This alignment is further supported by Narayan et al.'s (37). research on patient-centered care in home healthcare settings, which emphasizes the fundamental importance of relationship-building and comprehensive assessment skills. Their findings reinforce our observations about the critical role of open communication and collaborative approaches in creating an effective practice environment that supports person-centered practice delivery. The “Prerequisites” dimension of McCance and McCormack's model was strongly reflected in our implementation strategies. Our emphasis on continuous training and interprofessional relationships aligned with McCance and McCormack's focus on professional competence and interpersonal skills development. Quantitative data supported this alignment, with 67% of stakeholders strongly agreeing and 33% somewhat agreeing that they received adequate training, demonstrating robust commitment to professional development. These findings echo Watson's (23) research highlighting the critical importance of prerequisite interprofessional team skills in delivering person-centered practice.

Examining the organizational context more deeply reveals the critical role of macro-level context factors in enabling person-centered practices. Recent research by Roberts et al. (38) emphasizes the necessity of moving beyond theoretical PCC frameworks toward integrated care models for older adults, a perspective particularly relevant to our implementation context. The network demonstrated organizational readiness through three key elements: leadership commitment, organizational alignment, and resource allocation.

Leadership support emerged as a pivotal factor in implementing consistent person-centered values across geographically dispersed teams. Quantitative data underscored this support, with 83% of front-line staff strongly agreeing that branch managers supported the person-centered approach, and 67% strongly agreeing about overall management support. Branch managers and senior leadership played crucial roles in translating abstract person-centered principles into consistent operational practices, including unified approaches to documentation, care planning, and interdisciplinary collaboration. Without this leadership engagement, each branch would likely maintain its own distinct approach, perpetuating the coordination challenges the PPM sought to address. As one participant noted: “We have a management team right now that is supportive and encouraging.” This support extended beyond mere verbal endorsement to include practical implementation initiatives. Critically, the alignment between organizational mission and person-centered values proved crucial for implementation success. By incorporating the network's core values—responsibility, professionalism, and respect—into the PPM, the organization ensured cultural continuity and prevented common pitfall of implementing changes that conflict with existing organizational values.

Our in-depth examination of the data suggests that the combination of supportive leadership, aligned organizational values, and adequate resource allocation indicated a system well-prepared for the implementation of person-centered practices. Interestingly, our findings revealed that interprofessionalism received the lowest rating (3.7) among the identified values, despite being recognized as crucial for comprehensive care delivery. This apparent contradiction merits further examination. While care coordination and interprofessionalism are closely interconnected concepts, they were differentiated in our study—interprofessionalism focuses on collaborative practices among diverse professionals (knowledge sharing, mutual respect), while care coordination emphasizes the operational mechanisms that integrate these collaborative efforts into seamless service delivery. The lower rating of interprofessionalism may reflect the practical challenges in its implementation. This suggests that while stakeholders recognize the theoretical importance of interprofessional collaboration, they experience significant barriers to its practical application in daily operations. This finding aligns with research by Ashcroft et al. (39) highlighting the persistent challenges of establishing effective interprofessional practices in decentralized care systems, where geographical and organizational boundaries can impede collaborative relationships.

To address these interprofessional collaboration challenges, several strategies could be implemented in this decentralized home care context. First, establishing structured communication protocols specifically designed for geographically dispersed teams could facilitate more consistent information exchange. Regular interdisciplinary case conferences, both virtual and in-person, would create opportunities for meaningful collaboration despite physical separation. Additionally, implementing shared documentation systems accessible to all professionals involved in a patient's care would support timely information sharing. Finally, joint training initiatives focusing on collaborative competencies could help build the interprofessional relationships necessary for truly integrated care delivery. These approaches could help bridge the gap between the recognized importance of interprofessionalism and its practical implementation (40).

However, our findings also revealed certain implementation challenges that required attention. Team size affected communication effectiveness, with larger teams experiencing more difficulties in information exchange. Staff turnover complicated the maintenance of stable patient-provider relationships, whilst scheduling constraints and financial pressures created ongoing tensions between meeting patient preferences and maintaining organizational efficiency. These challenges highlighted the importance of maintaining focus on person-centered principles even in the face of operational pressures.

### Study limitations

Some limitations warrant consideration when interpreting our findings. The relatively small sample size of front-line staff in the implementation phase, whilst providing valuable insights, may not fully represent the diversity of perspectives within the organization. Additionally, our analysis lacks comprehensive demographic data about participants, including age, ethnicity, professional background, and years of experience—factors that could significantly influence perspectives on person-centered practice and serve as important cultural influences on the data collected.

Time constraints created periods of latency between study phases, potentially affecting participant engagement. Although these periods were managed through regular communication updates, they may have influenced the continuity of the development process. The potential for social desirability bias, particularly in focus groups and questionnaire responses, cannot be discounted despite efforts to ensure anonymity.

The study's single-organization focus, whilst allowing for depth of understanding, may limit the generalizability of findings to other home care contexts. Additionally, the study's timeframe did not permit long-term evaluation of implementation outcomes.

### Recommendations for home care management

By synthesizing our findings and translating them into practical implications, we propose several key recommendations for home care management. First, our findings highlight the crucial importance of leadership development in supporting person-centered practice implementation. Managers should receive specific training in person-centered leadership approaches, with particular focus on creating supportive practice environments. This training should encompass developing skills in facilitating open communication, managing diverse teams, and effectively balancing operational demands with person-centered principles. The establishment of regular leadership forums can help maintain consistency across branches whilst allowing for necessary local adaptation to specific contexts and needs.

Our research also emphasizes the need for robust structural support systems within organizations implementing person-centered practice. Healthcare organizations should establish clear mechanisms that support person-centered practice in daily operations. This includes ensuring dedicated time for team reflection and case discussions, which our findings showed were crucial for successful implementation. Organizations should develop flexible scheduling systems that can better accommodate patient preferences whilst maintaining operational efficiency (41). Additionally, implementing appropriate technology solutions can significantly enhance communication across geographically dispersed teams, addressing one of the key challenges identified in our study. Regular review mechanisms for person-centered practices ensure continuous alignment with organizational goals and patient needs.

Professional development emerged as a critical factor in successful implementation. Organizations should implement a comprehensive professional development framework that supports person-centered practice delivery. This framework should incorporate regular training in person-centered practice principles, ensuring all staff maintain current knowledge and skills. Mentorship programs for new staff have proved particularly valuable in transmitting person-centered values and practices. Creating opportunities for interprofessional learning can enhance collaboration and understanding across different professional groups, whilst recognition systems for person-centered practice excellence help reinforce desired behaviors and approaches (42).

Finally, our findings underscore the importance of robust evaluation and monitoring systems. Organizations should implement regular evaluation processes that assess the effectiveness of person-centered practice implementation. These evaluations should encompass patient experience measures to ensure care delivery aligns with patient needs and preferences. Regular staff satisfaction surveys can help identify areas requiring additional support or modification. Quality indicators aligned with person-centered principles provide objective measures of progress, whilst impact assessments of person-centered initiatives help demonstrate value and guide future developments. Together, these evaluation components create a comprehensive framework for monitoring and improving person-centered practice delivery in home care settings (43).

## Conclusion and future directions

This study makes a significant contribution to understanding how person-centered practice principles can be conceptualized and potentially applied in home care settings. Our findings demonstrate that a Professional Practice Model, when developed through collaborative engagement and aligned with established theoretical frameworks like McCance and McCormack's model, can provide a foundation for bridging the gap between theory and practice in home care delivery.

The alignment between our empirically developed model and McCance and McCormack's theoretical framework provides both validation and practical guidance for home care organizations seeking to implement person-centered approaches. This convergence suggests that person-centered practice principles are not just theoretically sound but could be practically achievable in-home care settings, given appropriate organizational support and implementation strategies.

Our findings illustrate a home care network with promising readiness for transformation. The PPM, informed by McCance and McCormack's model, provides a potential roadmap for transitioning person-centered practice from an aspirational ideal to an operational reality in home care settings. The model's potential to unify diverse practices across geographically dispersed teams suggests its possible value for other decentralized healthcare organizations.

Several areas warrant further investigation to build upon these findings. Longitudinal studies examining the implementation process and measuring outcomes if the PPM were to be fully implemented would provide valuable insights into the model's applicability and sustainability. Research exploring the development of person-centered practices in different cultural and organizational contexts could help identify universal principles and context-specific adaptations.

Additionally, investigation into the role of technology in supporting person-centered practice delivery in home settings could help address some of the communication and coordination challenges identified in our study. Research examining the economic implications of person-centered practice implementation would also be valuable for healthcare organizations considering similar transformations.

The journey toward truly person-centered home care continues to evolve. This study provides both theoretical insights and contextual analysis for organizations considering this transformation. As healthcare systems globally grapple with increasing demands and resource constraints, the importance of effective, person-centered approaches to home care delivery becomes ever more critical. Our findings suggest that with appropriate theoretical grounding, careful contextual analysis, and organizational support, such transformation may be possible and could potentially enhance both care delivery and professional practice in home care settings.

## Data Availability

The raw data supporting the conclusions of this article will be made available by the authors, without undue reservation.
